# Radical prostatectomy in metastatic prostate cancer: is there enough evidence? | *Opinion: No*

**DOI:** 10.1590/S1677-5538.IBJU.2016.05.05

**Published:** 2016

**Authors:** Benjamin T. Ristau, Marc C. Smaldone

**Affiliations:** 1 Division of Urologic Oncology, Fox Chase Cancer Center, Temple University Health System, Philadelphia, PA, USA

**Keywords:** Prostatectomy, Prostate, Prostatic Neoplasms

Despite an absence of level I data suggesting a survival benefit, interest in radical prostatectomy (RP) for patients with metastatic prostate cancer (PC) is rising (1). Traditionally, RP has been reserved for clinically localized PC, and good outcomes have been demonstrated in this population (2). While both retrospective and observational studies have reported improved survival outcomes for patients with metastatic (M1) disease who undergo primary tumor treatment relative to androgen deprivation therapy alone (1, 3), prospective data - particularly for surgery - is sparse. It would be unwise, then, to prematurely extrapolate these results to patients with metastatic disease until the merits of such an approach are carefully considered.

For the purposes of this discussion, we will consider metastatic disease to encompass both clinically node positive (cN1) and traditional metastatic (cM1) disease (4). There are no randomized trials exploring the role of RP in the cN1 setting. Thus, we are limited to retrospective analyses to inform treatment decisions. Though the relevance of the distinction has been recently questioned (5), the majority of these studies are comprised of patients with occult nodal disease (i.e. clinically unapparent and discovered at the time of radical prostatectomy) rather than clinical node positive disease (i.e. > 1cm nodes identified on pre-operative imaging studies). A German group looked at patients with cT1-3, N1-2, M0 prostate cancer who underwent pelvic lymph node dissection (PLND) with androgen deprivation therapy (ADT, group 1) versus RP+PLND+ADT (group 2) (6). Biochemical recurrence (BCR)-free survival, overall survival (OS), and prostate cancer specific mortality (PCSM) favored performance of RP. Unfortunately, marked differences in stage and grade also favored the RP group, thereby limiting the generalizability of these results. Similarly, Grimm and colleagues evaluated patients with node-positive PC who either underwent RP+ADT or ADT alone and demonstrated improved BCR-free survival and PCSM in the RP group (7). However, the group that was not selected for surgery had greater number of positive lymph nodes relative to the RP group, again highlighting selection biases inherent to this study design. In an attempt to account for these differences between groups, Ghavamian et al. matched 79 pN+ patients who underwent PLND and early adjuvant orchiectomy to 79 pN+ patients who underwent RP with PLND on the following parameters: number of positive nodes, clinical grade, clinical stage, patient age, year of surgery, and preoperative prostate-specific antigen (PSA) (8). Differences in OS at 10 years (66 vs. 28%; p < 0.001) and PCSM (21 vs. 61%; p <0.001) were noted; however, the observed survival benefits for the RP group were no longer apparent in a subset analysis of patients in the PSA-era. Considered in light of inherent biases and without the benefit of randomized trial data, the evidence in support of RP in the presence of positive lymph nodes is subjective at best.

Given the evidence supporting cytoreductive surgery in other malignancies and the increasing incidence of M1 PC in the United States (9-11), it is reasonable to consider the role of surgery as part of a multimodal treatment approach in these patients. The scientific rationale appears sound. Kaplan et al. have advanced the concept of the ‘premetastatic niche', whereby the primary tumor is the predominant source of metastasis through circulating tumor cells (12). There is also evidence supporting improved survival in preclinical models of M1 prostate cancer when the primary tumor is removed (13, 14). The current clinical evidence base for RP in M1 PC, however, is limited to retrospective analyses of administrative data sets. A Surveillance Epidemiology and End Results (SEER) study demonstrated decreased PCSM in patients undergoing either RP or brachytherapy (3). Similarly, an analysis of the National Cancer Database (NCDB) showed reduced overall mortality in all patients with treatment of the primary tumor, treatment effects that were most notable in healthy patients with lower risk tumors (15). Despite the large study population of these studies, they are nonetheless retrospective and still subject to coding errors and selection biases inherent to administrative datasets (16). Of five prospective clinical trials seeking to evaluate the role of primary tumor treatment in metastatic prostate cancer, two include a surgical arm (17). A multi-center North American trial (NCT01751438) is randomizing patients to best systemic therapy (BST) alone and BST plus definitive local therapy (radiation or surgery) in patients with metastatic PC. The primary outcome measure is progression-free survival defined as the time interval from the start of initial BST to the date of disease progression or death (whichever occurs first). The first interim analysis of NCT01751438 is near on the horizon, and initial results are expected in March 2018.

Despite enthusiasm for RP in metastatic PC, it is essential not to put the proverbial cart before the horse. The question is not only if RP has a role in metastatic PC, but also where in the disease process RP is most appropriate. The risk of local progression in systemically treated PC is not trivial. Reports of palliative cystoprostatectomy in patients with castration-resistant disease demonstrate substantially increased complication and reoperation rates (13% rectal injury, nearly 25% re-operation) compared to well established complication rates for prostatectomy performed in the setting of clinically localized disease (18, 19). In contrast, recently published data have demonstrated the feasibility and acceptable complication rates of RP performed for patients in the early metastatic setting (i.e. within 3-12 months of ADT ± systemic therapy initiation) (20). Although the most appropriate use of cytoreductive surgery may be within a multi-modal treatment algorithm early in the metastatic disease process, its optimal role in metastatic PC remains undefined.

The evidence for RP in metastatic prostate cancer is immature. While intriguing and hypothesis-generating, it is not yet robust enough to inform clinical decision-making, and surgery for metastatic disease is currently not included in contemporary best practice guidelines. Multiple trials are evaluating the role of local therapy (radiation and surgery) in conjunction with systemic therapy. Until these data are mature, the role of RP in metastatic prostate cancer is not ready for prime time and should only be explored in the context of a clinical trial.
